# Prevalence and Anatomical Distribution of Physiologic Gingival Pigmentation and Its Association With Facial Complexion and Lip Tone in a South Indian Population: A Cross-Sectional Analysis

**DOI:** 10.7759/cureus.104515

**Published:** 2026-03-01

**Authors:** Simran Chahal, Shankar S Menon, Maya Rajan Peter, Arun Kurumathur Vasudevan

**Affiliations:** 1 Department of Periodontics, Amrita School of Dentistry, Amrita Vishwa Vidyapeetham, Kochi, IND

**Keywords:** facial complexion, gingival pigmentation, lip tone, melanin, periodontal esthetics, south indian population

## Abstract

Background: Gingival pigmentation is a physiologic phenomenon primarily resulting from melanin deposition by melanocytes and demonstrates marked variation across ethnic groups due to genetic, environmental, and evolutionary influences. Despite India’s extensive phenotypic diversity, data on the prevalence and anatomic distribution of gingival pigmentation, particularly in South Indian populations, remain limited, even as interest in periodontal esthetics continues to increase.

Methodology: This cross-sectional study evaluated 100 systemically healthy individuals (54 male and 46 female individuals; mean age 38.31 ± 9.19 years) attending the Outpatient Department of Periodontics at the Amrita School of Dentistry, Kerala, between October 2024 and October 2025. Gingival pigmentation was assessed visually and classified. Lip pigmentation was assessed clinically using a descriptive visual grading based on the extent and intensity of melanin deposition on the vermilion border, and facial complexion was assessed using the Fitzpatrick scale adapted to the von Luschan chromatic scale. Descriptive statistics were used to assess prevalence and distribution patterns, while the Pearson chi-square test evaluated associations between gingival pigmentation and facial complexion, with significance set at p < 0.05.

Results: Class IV gingival pigmentation (moderate dark-hued islands) was the most prevalent pattern (37%), followed by Class VI (severe dark-hued islands, 21%) and Class I (absence of pigmentation, 20%). Facial complexion was predominantly mild brown (60%), and lip tones were most commonly brown (46%) or dark brown (44%). Statistical analysis revealed no significant association between gingival pigmentation and facial complexion (χ² = 13.643, df = 20, p = 0.848). Gender did not demonstrate any discernible influence on pigmentation patterns.

Conclusion: Moderate-to-severe physiologic gingival pigmentation is highly prevalent in South Indian individuals and appears to be independent of facial complexion and gender. These findings challenge assumptions of a direct phenotypic correlation between cutaneous and gingival pigmentation. While the results provide valuable insights for periodontal esthetic management, limitations, including the single-center design and reliance on visual assessment, warrant cautious interpretation. Future multicenter studies incorporating objective assessment tools are recommended to further elucidate gingival pigmentation patterns in diverse populations.

## Introduction

Gingival pigmentation arises from melanin synthesis by melanocytes located in the basal layer of the gingival epithelium, followed by the transfer of melanin granules to adjacent keratinocytes through a process termed the epidermal-melanin unit, as described by Nordlund [1]. For individuals with a prominent gingival display during smiling, called a "gummy smile," this physiological phenomenon has significant esthetic implications [2]. The color of the gingiva varies widely across the globe. While pale pink hues are typical in Caucasian populations, brown or blue-black colors are observed in individuals with African or Asian ancestry [3]. Such variation is generally attributed to differences in melanocyte density, activity, and melanin distribution within the oral mucosa [4].

India is a textbook study in genetic pigmentation, thanks to its huge ethnic and genetic diversity. India’s population exhibits a wide spectrum of skin tones. These range from fairer complexions in the northern parts of the country to darker shades toward the southern regions. This seems to have been shaped by historical migrations, genetic admixture, and environmental factors like exposure to ultraviolet radiation [5]. In particular, Indians living in the South seem to have darker skin tones, which resemble sub-Saharan African populations, in contrast to the lighter complexions seen among North Indians, which resemble European phenotypes [6]. Despite this diversity, the oral pigmentation patterns in Indian populations, especially in South India, have received limited attention in scientific literature.

Contemporary literature has studies on gingival pigmentation that have focused on other ethnic groups, namely the Nepalese and the African populations, and they have reported associations with skin color, gingival biotype, and lip tone [7,8]. Ponnaiyan et al.’s work in India suggested a plausible link between skin complexion and gingival pigmentation among South Indians. Their sample, however, was limited and lacked detailed distribution data. Since the demand for periodontal esthetic procedures is rising by the day, especially in techniques such as gingival depigmentation, as a result of patient dissatisfaction with pigmented gums, filling this knowledge gap becomes a matter of utmost importance [9,10]. Techniques such as scalpel surgery, cryotherapy, and laser ablation are well documented as procedures imperative toward reducing gingival pigmentation [11]. However, their success lies in understanding the prevalence of baseline pigmentation and its phenotypic correlation with specific populations.

This article addresses these deficiencies by calculating the prevalence and distribution of gingival pigmentation in an Indian population attending a tertiary care center in Kerala. This study also examines the correlations with facial complexion, lip tone, and gender by using standardized classification systems: Ponnaiyan et al. for gingival melanin distribution, descriptive visual grading for lip tone, and the Fitzpatrick scale for facial complexion [12]. This article attempts to improve upon clinical decision-making and esthetic outcomes in periodontal practice, particularly for patients seeking depigmentation therapies, by providing a detailed profile of gingival pigmentation in this hitherto understudied demographic. The present cross-sectional study was designed with the following objectives.

Primary objective

The primary objective is to determine the prevalence and anatomic distribution of physiologic gingival pigmentation in a South Indian population attending a tertiary care dental center.

Secondary objectives

The secondary objectives are to evaluate the association between gingival pigmentation and facial complexion, to descriptively assess lip pigmentation patterns within the study cohort, and to examine possible associations of gingival pigmentation with gender.

By clearly distinguishing prevalence estimation from phenotypic association analysis, this study aims to provide structured epidemiologic data while testing the commonly held assumption that gingival melanin deposition correlates directly with facial skin tone.

## Materials and methods

Study design

This was a cross-sectional study conducted at the outpatient department (OPD) of the Department of Periodontics, Amrita School of Dentistry, Kerala, India. Ethical approval was obtained from the Institutional Review Board of Amrita School of Medicine vide no. ECASM-AIMS-2024-093 dated February 20, 2024. Informed consent was obtained from the patients, who participated voluntarily and under the assurance of confidentiality.

Study population

The study participants were selected from patients who reported to the OPD between October 2024 and October 2025. The study employed a nonprobability convenience sampling technique. Inclusion criteria included systemically healthy individuals aged 18-50 years, irrespective of gingival pigmentation status. Both maxillary and mandibular gingiva were clinically examined during assessment. The highest observed pigmentation score across arches was recorded for classification. Exclusion criteria included individuals with systemic illnesses (e.g., diabetes and autoimmune disorders), current or past smoking habits, use of pigmentation-altering medications (e.g., minocycline), or prior gingival depigmentation procedures. These criteria ensured that physiologic pigmentation was focused on, unaffected by external confounding factors.

Examiner training and calibration

All clinical assessments were performed by a single calibrated examiner who is a qualified dental surgeon with postgraduate specialization in Periodontology. Prior to the commencement of data collection, the examiner underwent formal calibration under the supervision of a senior faculty member experienced in oral diagnosis and pigmentation assessment. Calibration involved reviewing the gingival pigmentation classification criteria, standardizing facial complexion categorization, and conducting a pilot examination of subjects not included in the final sample. To assess intraexaminer reliability, 30 participants were reevaluated after a one-week interval. Cohen’s kappa coefficient demonstrated substantial agreement (κ = 0.82), indicating consistent scoring reliability.

Clinical examination protocol

All examinations were performed in a standardized dental operatory setting under uniform light-emitting diode dental chair illumination. Participants were seated in an upright position, and soft tissue surfaces were gently air-dried prior to assessment. To minimize examiner fatigue and ensure scoring accuracy, no more than six to eight subjects were examined per day, clinical sessions did not exceed three hours per sitting, short breaks were incorporated after every 10 participants, and morning and afternoon sessions were alternated to avoid systematic bias.

Sample size calculation

The sample size was determined based on a previous study by Rijal et al., which reported a 49% prevalence of light brown gingival pigmentation in a comparable population [7]. Thereby, the minimum sample size was calculated as 100 participants and was deemed feasible within the one-year study timeframe.

The sample size was calculated using the single proportion formula: $n = Z^2 \times p \times (1-p) / d^2$.

where n is the required sample size, Z is the Z-score corresponding to 95% confidence level (1.96), p is the estimated prevalence (0.49, based on Rijal et al.), and d is the margin of error (0.10).

Upon substituting the values: n = (1.96)^2 ^× 0.49 × 0.51 / (0.10)^2^

The calculated minimum sample size was approximately 96, which was rounded to 100 participants.

Data collection procedures

A detailed oral examination was carried out. Gingival pigmentation was assessed visually and classified according to the Ponnaiyan et al. system, which divides it into six classes based on melanin distribution: Class I (no pigmentation), Class II (few light brown islands), Class III (mild dark-hued islands), Class IV (moderate dark-hued islands), Class V (dark-hued islands), and Class VI (severe dark-hued islands) [3]. Lip pigmentation was assessed clinically using a descriptive visual grading based on the extent and intensity of melanin deposition on the vermilion border, adapted from previously published oral pigmentation studies. Facial complexion was scored using the Fitzpatrick scale, adapted to the von Luschan chromatic scale, with categories from FC1 (creamy white, 1-2) to FC7 (intense dark, 31-36) [12]. Demographic details, including age and gender, were recorded via a structured pro forma. All data were anonymized to protect participant privacy. A full-mouth clinical examination was performed. However, pigmentation scoring was based primarily on the labial/buccal gingiva, as this region is most relevant esthetically and consistent with the Ponnaiyan classification system. All examinations were performed under standardized artificial dental operatory light, with participants seated in a dental chair in the OPD setting. No intraoral photographs were used for scoring or analysis. Clinical assessment was performed visually by the calibrated examiner.

Study tools

The classification systems were selected for their established use in oral and dermatologic research. The Ponnaiyan et al. system provided a scientific framework for gingival melanin distribution, while a descriptive visual grading based on the extent and intensity of melanin deposition on the vermilion border offered a granular assessment of lip pigmentation. The Fitzpatrick scale, widely validated for skin tone assessment, was adapted to ensure compatibility with the study’s Indian cohort [12].

Statistical analysis

Data were entered into Microsoft Excel (Microsoft Corporation, Redmond, WA) and analyzed using Statistical Package for the Social Sciences version 26.0 (IBM Corp., Armonk, NY). Descriptive statistics, including frequencies, percentages, means, and standard deviations, were used to summarize demographic variables, gingival pigmentation classes, lip tone categories, and facial complexion distribution. Normality of continuous variables (age) was assessed using the Shapiro-Wilk test prior to reporting the mean and standard deviation. As the age distribution did not significantly deviate from normality, parametric descriptive measures were considered appropriate. The association between gingival pigmentation and facial complexion was evaluated using the Pearson chi-square test. Prior to analysis, chi-square assumptions were assessed, including independence of observations and expected cell frequencies. It was observed that 73.3% of cells had expected counts less than 5, which may reduce the robustness of the chi-square approximation. Statistical significance was set at p < 0.05. Intraexaminer reliability for gingival pigmentation assessment was evaluated using Cohen’s kappa statistic, calculated from duplicate examinations of 15 participants at a two-week interval. Substantial agreement was observed (κ = 0.82), indicating acceptable reproducibility of clinical scoring. No formal post hoc power analysis was performed. Sample size was determined a priori using prevalence-based estimation with 95% confidence and a 10% margin of error.

## Results

A total of 100 participants (54 male and 46 female participants) were enrolled, with a mean age of 38.31 ± 9.19 years (range: 18-50 years). The study population reflected a balanced gender distribution and a broad age range, ensuring representativeness within the specified criteria.

Prevalence and distribution of gingival pigmentation

The distribution of gingival pigmentation was as follows: Class IV (moderate dark-hued islands) was the most prevalent (37%, n = 37), followed by Class VI (severe dark-hued islands, 21%, n = 21), Class I (no pigmentation, 20%, n = 20), Class V (dark-hued islands, 17%, n = 17), Class II (few light brown islands, 4%, n = 4), and Class III (mild dark-hued islands, 1%, n = 1). This pattern indicates a predominance of moderate-to-severe pigmentation, with a notable subset exhibiting no pigmentation, which is unexpected in a population typically associated with higher melanin expression.

Facial complexion distribution

Facial complexion was predominantly mild brown (60%, n = 60), followed by wheatish (17%, n = 17), moderate brown (17%, n = 17), fair (5%, n = 5), and dark brown (1%, n = 1). No participants exhibited creamy white (FC1) or intense dark (FC7) complexions, reflecting the intermediate to darker skin tones characteristic of South India.

Lip tone distribution

Lip tone was most commonly classified as brown (46%, n = 46) or dark brown (44%, n = 44), with light brown (7%, n = 7), pink lips (2%, n = 2), and chocolate brown (1%, n = 1) occurring less frequently. The absence of blackish-brown (LC6) lips suggests a ceiling effect on lip pigmentation severity within this cohort.

Association between gingival pigmentation and facial complexion

Cross-tabulation revealed varied distributions of gingival pigmentation across facial complexion categories (Table 1). For example, Class IV pigmentation was most common among mild brown complexions (56.8%, n = 21), while Class VI predominated in the same group (81.0%, n = 17). Conversely, fair complexions were rare across all classes, with only 5% of Class I and Class IV cases. The Pearson chi-square test showed no significant association between gingival pigmentation and facial complexion (χ² = 13.643, df = 20, p = 0.848). However, 73.3% of cells had expected counts less than 5, indicating potential statistical instability due to sparse data in some categories.

**Table 1:** Cross-tabulation of gingival pigmentation and facial complexion

Gingival pigmentation	Fair (%)	Whitish (%)	Mild brown (%)	Moderate brown (%)	Dark brown (%)	Total (n)
Class I	5.0	20.0	40.0	35.0	0.0	20
Class II	0.0	25.0	25.0	50.0	0.0	4
Class III	0.0	0.0	0.0	100.0	0.0	1
Class IV	5.4	21.6	56.8	13.5	2.7	37
Class V	11.8	11.8	76.5	0.0	0.0	17
Class VI	0.0	9.5	81.0	9.5	0.0	21
Total	5.0	17.0	60.0	17.0	1.0	100

Gender and age considerations

Gender distribution showed no marked influence on pigmentation patterns, with male (54%) and female (46%) participants exhibiting similar prevalence across classes. Age distribution was broad, with no apparent clustering of pigmentation severity by age group, suggesting that pigmentation is stable across adulthood in this population. Table 1 shows the cross-tabulation of gingival pigmentation and facial complexion as noted among the study participants.

## Discussion

This study offers a pioneering exploration of gingival pigmentation prevalence and distribution in an Indian population, revealing a high frequency of moderate-to-severe pigmentation (Classes IV and VI, 58% combined) at a tertiary care center in Kerala. These findings align with global observations that darker gingival tones predominate in populations of Asian and African descent, attributed to elevated melanocyte activity and melanin deposition in the oral mucosa [3]. However, the substantial proportion of Class I cases (20%), indicating no pigmentation, is striking in a cohort expected to exhibit widespread melanin expression based on regional skin tone trends [5]. This variability may reflect genetic heterogeneity in South India, where this study was conducted, with historical migrations and interbreeding producing a mosaic of phenotypic traits [6].

The absence of a significant association between gingival pigmentation and facial complexion (p = 0.848) contradicts prior studies suggesting a direct correlation between cutaneous and mucosal pigmentation [7,9]. For instance, Rijal et al. found a stronger association in a Nepalese population, in which lighter skin tones were associated with reduced gingival pigmentation [7]. Similarly, Ponnaiyan’s earlier South Indian study hinted at a relationship, though its smaller sample and lack of statistical rigor limited its conclusiveness. The current results suggest that gingival melanin expression may be governed by distinct regulatory mechanisms, potentially involving localized melanocyte-keratinocyte interactions rather than systemic melanin production tied to skin complexion [1]. Environmental factors, such as chronic sun exposure common in Kerala’s tropical climate, could also influence cutaneous pigmentation independently of the gingiva, further decoupling these traits [13,14].

The predominance of mild brown complexions (60%) and brown-to-dark-brown lip tones (90% combined) aligns with South India’s phenotypic profile, yet the lack of correlation with gingival pigmentation raises questions about the utility of facial esthetics as a predictor in clinical settings. Lip tone, while not statistically analyzed for association due to study design limitations, exhibited a distribution that paralleled facial complexion, suggesting a possible shared melanin-regulation pathway in these tissues. Future studies should incorporate lip tone into association analyses, as it may influence patient perceptions of oral esthetics and treatment expectations.

Gender exhibited no influence on pigmentation patterns, consistent with prior research indicating that gingival pigmentation is not sexually dimorphic [2]. The broad age range (18-50 years) and lack of age-related trends further suggest that pigmentation remains stable across adulthood, likely reflecting a genetically determined trait rather than an age-dependent process. However, longitudinal studies could clarify whether subtle changes occur over time, particularly in response to environmental stressors.

Clinically, these findings have significant implications for periodontal esthetic management. The high prevalence of Class IV and VI pigmentation indicates that many South Indian patients may seek depigmentation for cosmetic reasons, particularly those with gummy smiles [10]. Techniques such as laser therapy or surgical excision, while effective, must be tailored to individual pigmentation severity and patient expectations [11]. The independence of gingival pigmentation from skin complexion challenges clinicians to rely on intraoral assessments rather than external phenotypic cues when planning interventions. Moreover, the presence of Class I cases suggests that not all patients will require treatment, highlighting the importance of patient consultation to align procedures with esthetic goals.

Limitations

Several limitations must be considered when interpreting the findings of this study. First, the study employed a single-center, nonprobability convenience sampling design conducted at a tertiary care institution in Kerala. Although the cohort represents a South Indian population, the findings cannot be generalized to the entire Indian subcontinent, which exhibits substantial regional, ethnic, and genetic heterogeneity [5,6]. This sampling method may limit generalizability beyond the studied tertiary care population and may introduce selection bias. A multicentric study incorporating northern, southern, eastern, and western populations would provide broader representativeness and allow interregional comparisons.

Second, gingival pigmentation, lip tone, and facial complexion were assessed using visual clinical examination performed by a single calibrated examiner under standardized dental operatory LED lighting conditions. Although intraexaminer reliability demonstrated substantial agreement (κ = 0.82), visual assessment remains inherently subjective and susceptible to measurement bias [1,3]. Objective methods such as reflectance spectrophotometry, digital colorimetric analysis, or standardized photographic calibration were not utilized, which may limit reproducibility and precision [11].

Third, facial complexion was categorized using the Fitzpatrick skin phototype scale adapted to correspond with von Luschan chromatic groupings [12]. However, physical von Luschan chromatic tiles were not employed, and classification was based on visual alignment with standardized descriptors. While clinically practical, this approach may reduce interstudy reproducibility.

Fourth, lip pigmentation assessment was descriptive and exploratory in nature. Although derived from previously published descriptive frameworks [8], it does not represent a universally validated standardized index. While lip tone was recorded to provide additional phenotypic context, inferential statistical analysis involving lip pigmentation was not performed due to sample size constraints and sparse category distribution. Future studies with larger samples should incorporate lip tone into multivariable association models to better understand mucocutaneous pigmentation relationships.

Fifth, chi-square analysis evaluating the association between gingival pigmentation and facial complexion demonstrated a high proportion of cells with expected counts below five (73.3%). This exceeds conventional assumptions for valid chi-square approximation, which recommend that no more than 20% of cells have expected counts less than five and that no cell have an expected count below one. Consequently, the statistical inference derived from this test may be unstable, and the reported p value should be interpreted with caution. The possibility of a Type II error cannot be excluded. A larger sample size or alternative statistical approaches, such as category consolidation or exact testing methods, may enhance analytical stability and provide a more reliable assessment of associations [7].

Sixth, no multivariable analysis was performed to adjust for potential confounding variables. Although the primary objective of the study was descriptive (prevalence and distribution), the absence of multivariate modeling limits the ability to control for interacting factors that may influence pigmentation patterns. Future investigations incorporating regression-based analytical frameworks would provide a more rigorous evaluation of phenotypic associations [7].

Seventh, smokers and individuals with systemic illnesses were excluded to isolate physiologic pigmentation patterns. While methodologically appropriate for internal validity, this exclusion limits applicability to broader clinical populations where such variables are common [2].

Eighth, potential confounding variables, including gingival biotype, oral hygiene status, cumulative sun exposure, socioeconomic background, and genetic determinants of melanin expression, were not assessed or controlled for in the present cross-sectional design [4,14]. The absence of multivariable adjustment may limit the interpretation of phenotypic associations.

Finally, the cross-sectional design precludes causal inference and does not allow evaluation of longitudinal changes in pigmentation patterns across the lifespan [12].

Future investigations should adopt multicenter designs, larger and more regionally diverse samples, randomized sampling strategies, objective pigmentation assessment tools, and multivariable analytical approaches to further elucidate determinants of gingival pigmentation in the Indian population.

Future perspectives

Future research should prioritize multicenter studies incorporating populations from northern, southern, eastern, and western regions of India to better capture the country’s substantial genetic and phenotypic heterogeneity [5,6]. Larger, probabilistic sampling strategies would enhance representativeness and reduce selection bias, thereby strengthening external validity and enabling more robust interregional comparisons.

The incorporation of objective pigmentation assessment tools is strongly recommended. Techniques such as reflectance spectrophotometry and digital colorimetric analysis have demonstrated utility in quantifying melanin distribution in cutaneous tissues [11] and may improve precision and reproducibility when adapted for intraoral use. Development of a standardized, quantitatively validated gingival pigmentation index would facilitate cross-study comparability.

Future investigations should also employ multivariable analytical models to account for potential confounders. Variables such as gingival biotype, oral hygiene status, cumulative ultraviolet exposure, and socioeconomic determinants may influence pigmentation expression. Exploration of genetic determinants, including polymorphisms in melanogenesis-regulating genes such as MC1R, may further clarify biologic mechanisms underlying mucosal pigmentation variability [14]. Integrating clinical phenotyping with molecular insights could advance understanding beyond descriptive epidemiology. Longitudinal cohort designs would help determine whether gingival pigmentation remains stable across adulthood or demonstrates subtle temporal variation. Inclusion of broader clinical populations, including smokers and medically controlled patients, in stratified analyses may also improve clinical applicability while preserving internal validity.

Equally important is the incorporation of patient-centered research. Qualitative studies assessing patient perception of gingival pigmentation, esthetic expectations, and quality-of-life impact could guide ethical and individualized depigmentation protocols. Comparative outcome studies evaluating different depigmentation techniques in relation to baseline pigmentation severity may further refine evidence-based periodontal esthetic management [10].

This study highlights the complexity of gingival pigmentation in a South Indian cohort and demonstrates a high prevalence of moderate-to-severe physiologic forms, without significant association with facial complexion. These findings challenge assumptions of predictable phenotypic correlation and reinforce the importance of objective intraoral assessment. Collectively, they provide a foundation for future multicentric, biologically integrated, and patient-centered research aimed at optimizing aesthetic periodontal care.

## Conclusions

This cross-sectional study demonstrates that moderate-to-severe physiologic gingival pigmentation (Classes IV and VI) is highly prevalent within this South Indian cohort, with 58% of participants exhibiting these patterns. A notable proportion (20%) presented with no clinically appreciable pigmentation (Class I), underscoring phenotypic variability within the population. Within the constraints of the present sample and analytical framework, no statistically demonstrable association was identified between gingival pigmentation and facial complexion. However, given the high proportion of contingency table cells with low expected counts and the exploratory nature of the inferential analysis, these findings should be interpreted with caution. The study was primarily designed and powered to estimate prevalence and distribution patterns rather than to establish definitive phenotypic correlations.

The descriptive findings contribute region-specific baseline data on gingival pigmentation patterns in a South Indian population and highlight the importance of individualized intraoral assessment rather than reliance on external phenotypic cues alone. While the results do not support a clear association between cutaneous complexion and gingival pigmentation in this cohort, larger multicentric studies with more robust statistical modeling are required before broader conclusions can be drawn. Overall, this study provides foundational epidemiologic insight into physiologic gingival pigmentation in a defined population and offers a platform for future research incorporating larger samples, objective pigmentation assessment tools, and multivariable analytical approaches to refine understanding and support evidence-based esthetic periodontal care.

## References

[1] Nordlund JJ (2007). The melanocyte and the epidermal melanin unit: an expanded concept. Dermatol Clin.

[2] Dummett CO, Barens G (1967). Pigmentation of the oral tissues: a review of the literature. J Periodontol.

[3] Ponnaiyan D, Jegadeesan V, Perumal G, Anusha A (2014). Correlating skin color with gingival pigmentation patterns in South Indians - a cross sectional study. Oral Health Dent Manag.

[4] Jablonski NG, Chaplin G (2000). The evolution of human skin coloration. J Hum Evol.

[5] Stokowski RP, Pant PV, Dadd T (2007). A genomewide association study of skin pigmentation in a South Asian population. Am J Hum Genet.

[6] Reich D, Thangaraj K, Patterson N, Price AL, Singh L (2009). Reconstructing Indian population history. Nature.

[7] Rijal A, Dhami B, Pandey N, Aryal D (2021). Prevalence of gingival pigmentation and its association with gingival biotype and skin colour. J Nepal Soc Periodontol Oral Implantol.

[8] Hedin CA, Axéll T (1991). Oral melanin pigmentation in 467 Thai and Malaysian people with special emphasis on smoker's melanosis. J Oral Pathol Med.

[9] Deepak P, Sunil S, Mishra R, Sheshadri Sheshadri (2005). Treatment of gingival pigmentation: a case series. Indian J Dent Res.

[10] Kumar S, Bhat GS, Bhat KM (2012). Development in techniques for gingival depigmentation - an update. J Indian Soc Periodontol.

[11] Goldzieher JW, Roberts IS, Rawls WB, Goldzieher MA (1951). Chemical analysis of the intact skin by reflectance spectrophotometry. AMA Arch Derm Syphilol.

[12] Fitzpatrick TB (1988). The validity and practicality of sun-reactive skin types I through VI. Arch Dermatol.

[13] Diffey BL (1991). Solar ultraviolet radiation effects on biological systems. Phys Med Biol.

[14] Rees JL (2003). Genetics of hair and skin color. Annu Rev Genet.

